# The osteocutaneous superficial circumflex iliac artery (SCIP) flap in extremity reconstruction

**DOI:** 10.1016/j.jham.2025.100337

**Published:** 2025-08-05

**Authors:** Cédric Zubler, Mihai A. Constantinescu, Ioana Lese, Radu Olariu

**Affiliations:** Department of Plastic and Hand Surgery, Inselspital University Hospital Bern, University of Bern, Freiburgstrasse 18, 3010, Bern, Switzerland

**Keywords:** Superficial circumflex iliac artery flap, SCIP, Vascularized bone transfer, Extremity reconstruction, Orthoplastic, Perforator flap

## Abstract

**Introduction:**

Reconstruction of composite defects involving both soft tissue and bone in the extremities remains a complex challenge in reconstructive surgery. The osteocutaneous superficial circumflex iliac artery perforator (SCIP) flap combines a pliable skin island with vascularized iliac bone, offering a potential solution. However, reports on its application in reconstruction of the upper and lower limb remain limited. This study evaluates our clinical experience using osteocutaneous SCIP flaps for extremity reconstruction, with particular attention to surgical details, bony union and long-term outcomes.

**Methods:**

A retrospective review was conducted of all patients who underwent upper or lower extremity reconstruction with an osteocutaneous SCIP flap between September 2019 and April 2024 at a single tertiary trauma centre. Clinical data, surgical details, complications, and follow-up outcomes were collected. Bone union was assessed radiographically, and functional outcomes were evaluated using the Lower Extremity Functional Scale (LEFS) where applicable.

**Results:**

Nine patients (eight male, one female; mean age 48 years) underwent reconstruction using the osteocutaneous SCIP flap - six in the lower limb and three in the upper extremity. All flaps survived, providing successful soft tissue coverage. Full-thickness iliac bone segments (mean 5 × 3.2 cm) were harvested. Bony union was achieved in 8 of 9 cases (89 %) after a mean of 8.25 months. One case of pseudoarthrosis required secondary bone grafting. Two early postoperative hematomas were surgically drained, and one patient developed a donor-site iliac wing fracture, managed conservatively. Functional outcomes were favourable: all lower limb patients achieved full weight-bearing ambulation (mean LEFS score 59.4), and upper extremity patients regained useful hand function. Mean postoperative follow-up was 26.3 months.

**Conclusion:**

The osteocutaneous SCIP flap is a reliable option for reconstruction of composite defects in the extremities, offering stable soft tissue coverage and vascularized bone suitable for structural support and osseous integration. In our opinion, this flap represents a valuable addition to the reconstructive toolbox, particularly in cases requiring a moderately sized segment of bone and thin, customizable soft tissue coverage.

## 1 Introduction

Reconstruction of complex composite defects involving both soft tissue and bone in the extremities remains a significant challenge in reconstructive surgery. These injuries often result from high-energy trauma, severe infection, or oncologic resection and require a reconstructive approach that provides reliable soft tissue coverage of vital structures as well as structural support to restore function and prevent complications such as chronic osteomyelitis or non-union. In this context, free tissue transfer has nowadays become the gold standard to address many of these defects.

First described by Koshima et al., in 2004, the superficial circumflex iliac artery perforator (SCIP) flap has been recognised for overcoming some of the limitations associated with the classical groin flap - such as its bulk and short pedicle length.^1^ Since then, the SCIP flap has gained increasing popularity in reconstructive units all over the world. As anatomical understanding and surgical expertise with the SCIP flap have evolved, various modifications have been introduced, including adjustments to the plane of elevation, pedicle dissection, and tissue components included in the flap.^2^, ^3^, ^4^, ^5^ Initially introduced as a purely fasciocutaneous flap, alternative designs of the flap now offer the possibility to incorporate various other tissues such as lymph nodes, nerve, fascia or iliac bone.^6^

The concept of harvesting vascularized iliac bone dates back to 1978, when Taylor et al. described a composite groin flap incorporating iliac crest bone.^7^ This initial description was based on the superficial circumflex iliac artery (SCIA) system, however, subsequent preference was given to the deep circumflex iliac artery (DCIA) design due to the more sizable pedicle and its ability to support a larger bone segment.^8^ Interest in SCIA-based osteocutaneous flaps was revived in 2013 with the reintroduction of the osteocutaneous SCIP flap, which integrated the benefits of a thin, pliable skin island with a vascularized segment of iliac bone.^9^

Historically, vascularized iliac bone has been widely used in maxillofacial reconstruction - particularly for mandibular defects - owing to its naturally matching curvature and tricortical bone, which provides reliable support for prosthetic implants.^10^^,^^11^ Accordingly, the osteocutaneous SCIP flap has similarly been employed in this setting.^9^^,^^12^ However, evidence on the use of osteocutaneous SCIP flaps for reconstruction of composite defects in the extremities is more limited, especially in the western population. Furthermore, much of the existing literature focuses on surgical descriptions and flap survival rather than long term outcomes such as bony consolidation and potential secondary complications.^13^^,^^14^ The literature of the most recent years mainly shows single patient case reports on the experience of using the osteocutaneous SCIP for tibial reconstruction after chronic osteomyelitis^15^ or for reconstruction following significant trauma to the foot.^16^^,^^17^

This study aims to present our clinical experience with the osteocutaneous SCIP flap in reconstruction of composite defects of the upper and lower extremities. We describe the indications, technical nuances, and outcomes in a consecutive series of patients, highlighting the flap's utility, anatomical considerations, and potential role within the broader reconstructive algorithm.

## 2 Methods

A retrospective review was conducted at a single tertiary trauma centre, evaluating all free flap procedures performed between September 2019 and April 2024. This timeframe was chosen because we first used the osteocutaneous SCIP flap in 2019, while the endpoint allows for a minimum postoperative follow-up duration of one year. All patients who underwent reconstructions of upper or lower extremity defects using a free osteocutaneous SCIP flap during this period in our unit were included. No exclusion criteria were applied, and no patients were excluded.

Clinical and surgical data were retrieved from the hospital's electronic medical records system. The primary outcomes of interest included flap survival, overall reconstructive success, bone union, postoperative complications, and any secondary surgical interventions. Additional data on intraoperative technique and anatomy, the number and nature of prior procedures as well as their respective complications, and postoperative rehabilitation were collected from operative reports and follow-up documentation. Serial radiographs and, where indicated by the anatomical site and complexity of the defect, computed tomography, were used to judge osseous union. In cases where a specific outcome measure was unavailable, this patient's data was excluded from the final analysis of the respective outcome. To minimize bias, case files and imaging studies were independently reviewed by a reconstructive surgeon not involved in the original procedures.

As per our institutional protocol, patients undergoing free flap reconstruction are followed at standardized intervals: 1 week, 3 weeks, 2 months, 6 months, and one year postoperatively, with additional follow-ups arranged as clinically indicated. At the time of data collection, no patients have been lost to follow-up.

This study received approval from the local ethics committee (BASEC-Nr: Req-2022–00179), and reporting followed the STROBE (Strengthening the Reporting of Observational Studies in Epidemiology) guidelines for cohort studies.

## 3 Results

A total of nine consecutive patients underwent reconstruction of composite soft tissue and osseous defects in the extremities using osteocutaneous superficial circumflex iliac artery perforator flaps during this time. Of these, six involved the lower limb and three the upper extremity, as outlined in Table 1. The cohort consisted of eight male and one female patient, with a mean age at the time of reconstruction of 48 years (range: 26–66 years) and a mean body mass index (BMI) of 25.1 kg/m^2^ (range: 19.6–30.9 kg/m^2^).

**Table 1:** Summary of patient demographics and preconditions prior to reconstruction.

Case Number	Age at surgery	Sex	BMI (kg/m2)	Indication	Location of bone defect	Time between trauma and SCIP-reconstruction (days)	Number of local operations before SCIP-reconstruction	Clinically relevant local infection
1	31	Male	25	Acute trauma (shotgun injury)	Calcaneus	17	5	No
2	61	Male	31	Acute trauma (circular saw injury)	Metacarpal bones	2	1	No
3	64	Female	20	Acute trauma (lawnmower injury)	Metatarsal bone	9	3	No
4	26	Male	21	Chronic sequelae of trauma (crushing injury)	Metacarpal bones	583	9	yes
5	54	Male	28	Chronic sequelae of trauma (skiing accident)	Tibia	940	5	yes
6	33	Male	24	Chronic sequelae of trauma (skiing accident)	Tibia	238	12	yes
7	64	Male	26	Acute trauma (fall injury)	Calcaneus	22	7	yes
8	66	Male	30	Chronic sequelae of trauma (work accident)	Tibia	1944	8	yes
9	33	Male	21	Chronic sequelae of trauma (work accident)	Humerus	118	8	yes

All reconstructions were performed in the context of traumatic injuries, four in an acute setting, while five addressed chronic sequelae such as chronic osteomyelitis or pseudoarthrosis. In the acute setting, the definitive reconstruction using an osteocutaneous SCIP flap was performed on average 12.5 days (range 2–22 days) after the initial injury and following a mean of four (range 1–7) prior local operations. In contrast, reconstructions in the chronic group were performed at a mean interval of 764.6 (range 118–1944 days) days post-injury and after an average of 8.4 (range 5–12) previous operations. Clinically relevant surgical site infections were present in six (67 %) patients prior to definitive reconstruction (See Table 1).

### 3.1 Surgical details

**Fig. 1:**
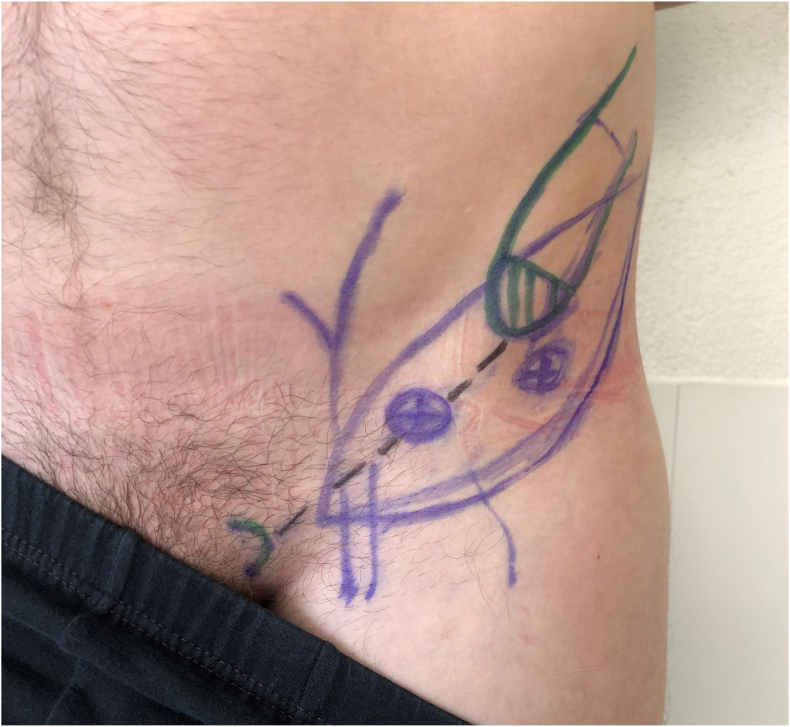
Example of the preoperative markings used for a chimeric osteocutaneous SCIP flap based on the deep branch. The planned skin paddle is outlined in blue, including the location of the relevant perforators as identified by duplex ultrasound. Furthermore, regional superficial veins in the medial aspect of the flap are also marked in blue. The green markings show the pubic tubercle (medial) and the iliac crest (lateral). The first 2 cm of the anterior superior iliac spine are dashed in green as bone harvest will only be commenced posterior to that in order to leave the origin of the inguinal ligament and the sartorius muscle in place. The black line represents the inguinal ligament.

A detailed description of the surgical technique used for flap planning and harvest has previously been published.^18^ An example of the preoperative markings is displayed in Fig. 1. All flaps were elevated on the superficial plane, based primarily on perforators from the deep branch of the SCIA, perforators of which were used for the perfusion of soft tissue component in all cases. In three cases, additional perforators from the superficial branch were included to ensure adequate vascularization, especially in larger flaps. Overall, the skin paddle used measured a mean length of 17 cm (13–22 cm) and width of 7.2 cm (range 5–9 cm), resulting in an average flap size of 125.1 cm^2^ (range 65–180 cm^2^). The perfusion of the bone component also originated from the deep branch in all cases. Full thickness bone segments were harvested from the iliac crest, with mean dimensions of 5 cm (range 3–7 cm) in length and 3.2 cm (range 2–4 cm) in height.

Usually, the entire length of the SCIA was dissected, harvesting it directly at the origin from the femoral artery, which resulted in a mean diameter of the arterial pedicle of 2.3 mm (range 1.5–3 mm). Arterial inflow was established primarily via end-to-side anastomoses. End-to-end anastomosis to a major vessel was only performed in one case where the recipient vessel had already been disrupted during initial trauma.

To enhance venous drainage, we routinely include a superficial vein found in the medial aspect of the flap, allowing for two separate venous anastomoses. Only one flap relied exclusively on the accompanying vena comitans of the SCIA. This vena comitans had a mean diameter of 1.6 mm (1–2.5 mm), whereas the superficial vein had an average diameter of 2.7 mm (2–3.5 mm). All venous anastomoses were performed in an end-to-end technique using couplers (See Table 2).

**Table 2:** Technical details related to the osteocutaneous SCIP flap used for reconstruction. SCIA = superficial circumflex iliac artery.

Case Number	Size of skin paddle (cm)	Size of bone segment (cm)	SCIA branch used for bone perfusion	SCIA branch used for skin perfusion	Plane of elevation	Diameter arterial pedicle (mm)	Diameter vena comitans (mm)	Diameter superficial vein (mm)	Recipient vessel	Suture technique	Number of venous anastomoses performed
1	16 x 7	7 x 3	Deep branch	Deep and superficial branch	Superficial plane	2.2	1.5	2	A. tibialis posterior	End-to-side	2
2	16 x 6	5 x 4	Deep branch	Deep and superficial branch	Superficial plane	2.3	1.5	3	A. ulnaris	End-to-side	2
3	13 x 7	4 x 2	Deep branch	Deep branch	Superficial plane	1.5	1	2.5	A. dorsalis pedis	End-to-side	2
4	13 x 5	4 x 3	Deep branch	Deep branch	Superficial plane	2	1.5	2	A. radialis	End-to-end	2
5	20 x 8	5.5 x 3	Deep branch	Deep and superficial branch	Superficial plane	1.8	1	2.5	A. tibialis posterior	End-to-side	2
6	15 x 8	6 x 3	Deep branch	Deep branch	Superficial plane	2	2	2.5	A. tibialis posterior	End-to-side	2
7	20 x 9	6 x 4	Deep branch	Deep branch	Superficial plane	3	2	3.5	A. fibularis	End-to-side	2
8	22 x 8	4.5 x 3.5	Deep branch	Deep branch	Superficial plane	3	1.5	3	A. tibialis anterior	End-to-side	1
9	18 x 7	3 x 3	Deep branch	Deep branch	Superficial plane	2.8	2.5	3.5	A. Brachialis	End-to-side	2

### 3.2 Complications, follow-up and bony consolidation

At the recipient site we experienced two hematomas that required surgical drainage in the early postoperative phase. Otherwise, all flaps survived, providing adequate soft tissue coverage. The donor sites all healed uneventfully, apart from one patient who developed a non-displaced iliac wing fracture as a complication of the bone harvest, which was treated conservatively.

After definitive flap reconstruction, patients stayed hospitalized for an average of 15.9 days. However, unsurprisingly, there was a significant difference between the upper and lower extremity cases, with the latter leaving the hospital significantly later (19.2 days versus 9.3 days, p-value <0.02) due to associated injuries, postoperative bed rest and established dangling protocols.^19^

Later on, one patient in which the flap covered part of the weight bearing aspect of the heel developed a wound dehiscence after returning to full weight bearing, resulting in revision and secondary wound closure 19 weeks after initial flap reconstruction. With appropriate offloading and the subsequent use of customized orthopaedic insoles, soft tissue coverage has been stable ever since.

**Fig. 2:**
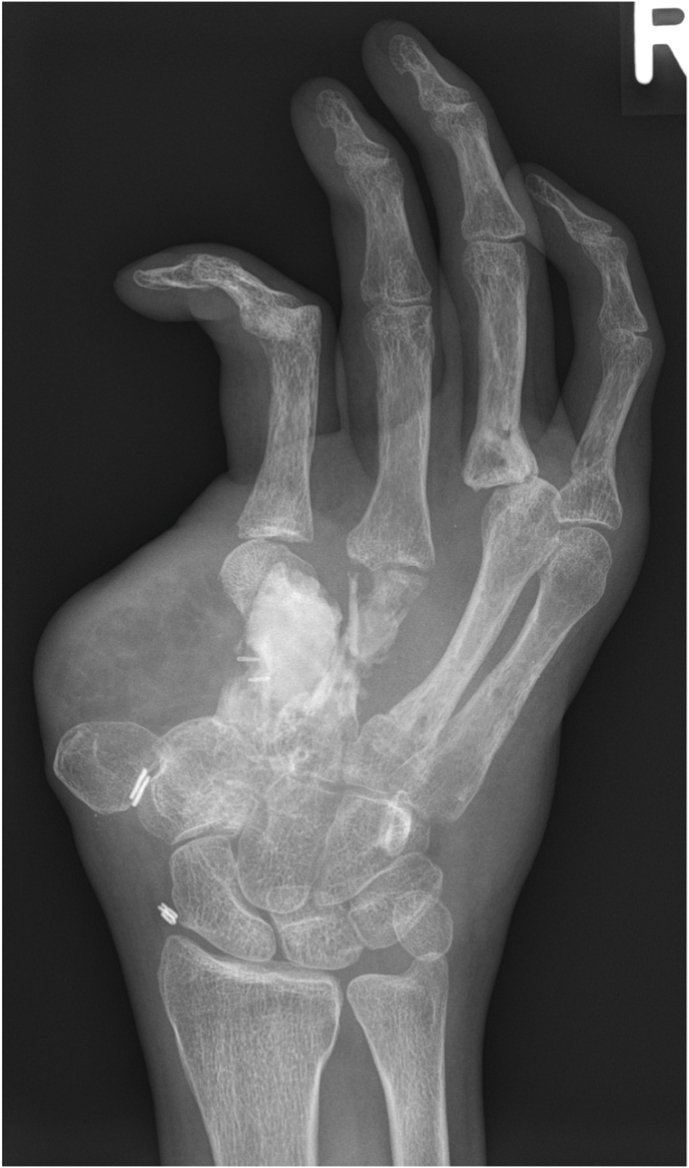
Pre-reconstruction radiograph 1.5 years after severe, devascularizing trauma to the right hand and multiple previous operations, resulting in osteonecrosis and collapse of the second and third metacarpal rays, despite the attempt to maintain length by using a bone cement spacer.

**Fig. 3:**
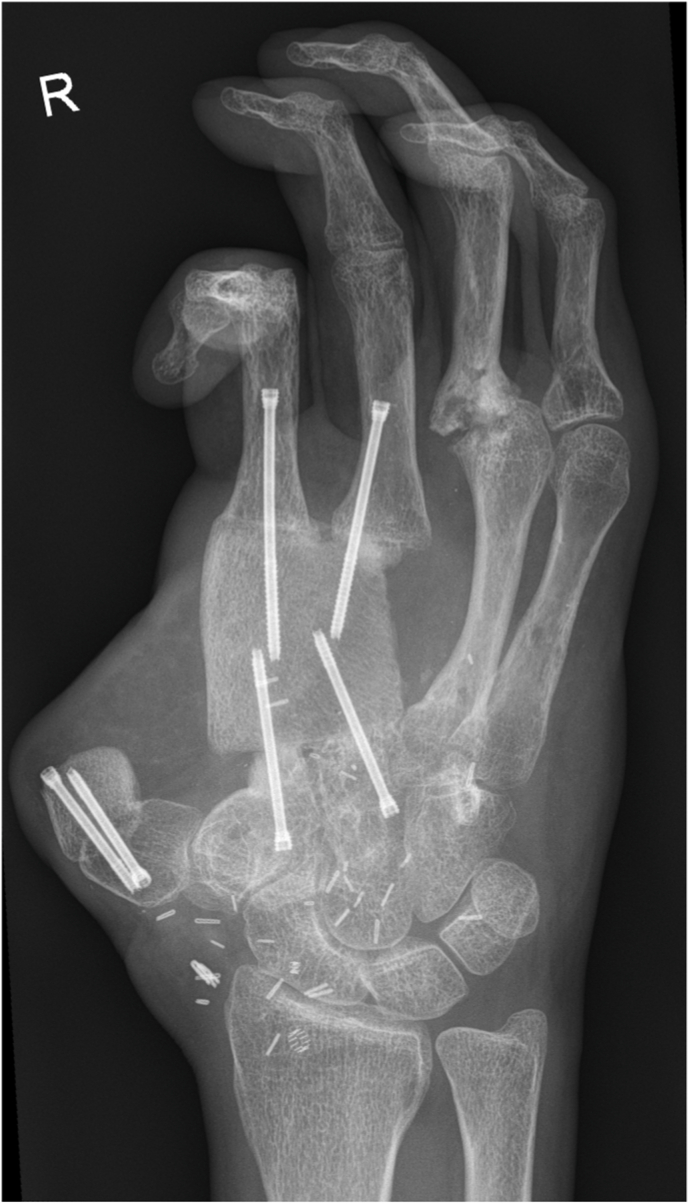
Early postoperative radiograph after reconstruction with an osteocutaneous SCIP flap, replacing the second and third metacarpal bones.

**Fig. 4:**
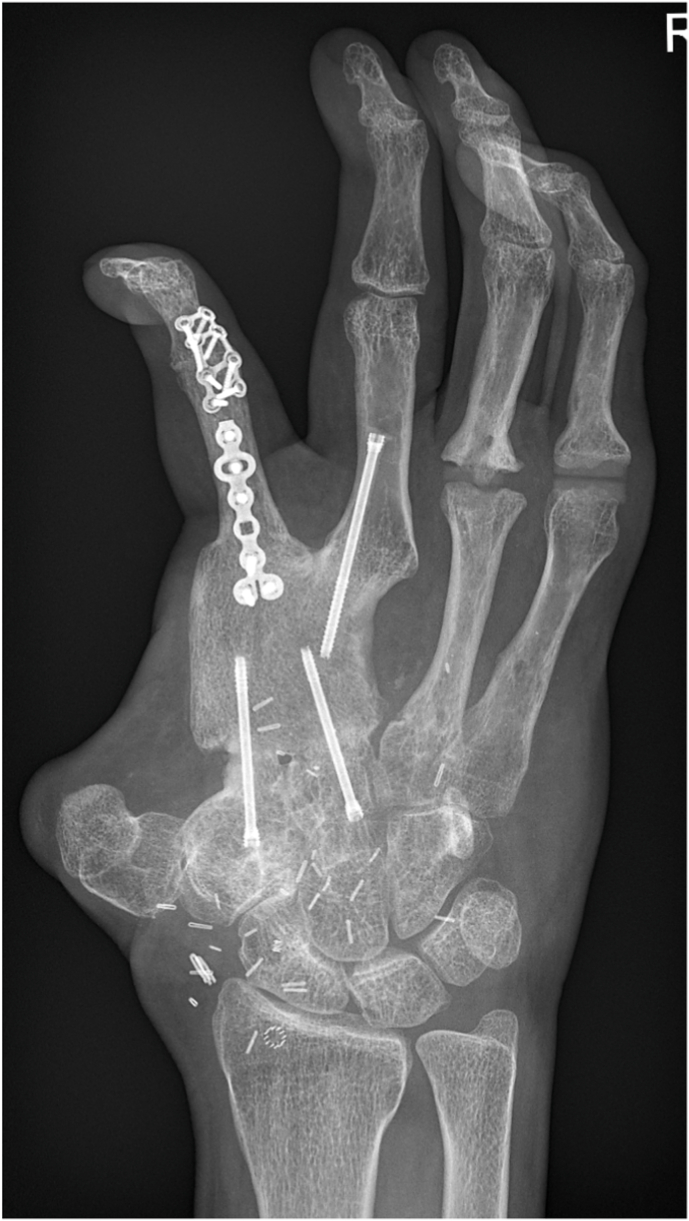
Late postoperative radiograph 2.5 years post-reconstruction, illustrating complete bony consolidation and progressive remodelling of the bone.

Complete bony union was achieved in 89 % of cases after a mean postoperative follow-up of 8.25 months (range 4–12 months). An example of the perioperative bony development is illustrated in Fig. 2, Fig. 3, Fig. 4. One lower limb patient developed pseudoarthrosis at the docking site of the posterior end of the iliac bone segment, while the anterior side healed completely. Revision of the pseudoarthrosis and bone grafting was undertaken 25 months after the initial reconstruction. Furthermore, both hand reconstruction cases underwent secondary procedures to improve overall function, such as removal of osteosynthesis material, tenolysis, arthrolysis or arthroplasty.

All lower extremity patients achieved unassisted, full weightbearing ambulation with a mean of 59.4 points (range 50–72 points) in the Lower Extremity Functional Scale. In the upper extremity patients, functional outcomes varied greatly depending on the initial injury, however, they all regained useful function for daily activities. Overall, the mean postoperative follow-up was 26.3 months (range 13–42 months) (See Table 3).

**Table 3:** Overview of complications, secondary procedures and postoperative follow-up. MCP = metacarpophalangeal joint.

Case Number	Flap-related complications	Hospital stay after SCIP reconstruction (days)	Time to union on X-ray (months)	Bony union (complication)	Secondary orthopaedic procedures	Postoperative Follow-up (months)
1	Late wound dehiscence	15	12	None	None	38
2	None	8	8	None	Hardware removal, arthrolysis MCP III + V, arthroplasty MCP IV, tenolysis extensor tendons III-V	24
3	None	13	10	None	None	36
4	Hematoma	7	4	None	Hardware removal, correction osteotomies, arthroplasty	42
5	None	14	12	None	None	38
6	Iliac wing fracture	30	–	Pseudoarthrosis	Revision of pseudoarthrosis, bone-grafting	17
7	None	21	8	None	None	16
8	Hematoma	22	6	None	None	13
9	None	13	6	None	None	13

### 3.3 Case example

**Fig. 5:**
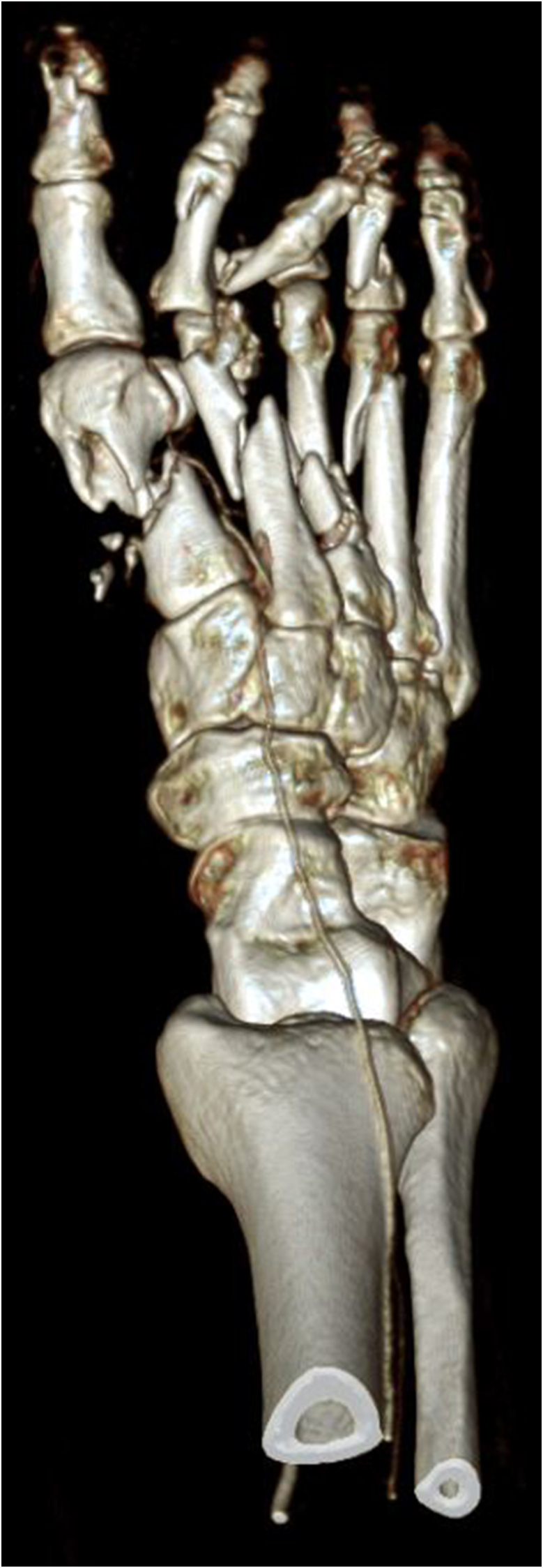
Initial 3D CT reconstruction of the affected right foot, illustrating multiple fractures of both metatarsal and phalangeal bones as well as the bone loss and shortening of the first metatarsal ray.

**Fig. 6:**
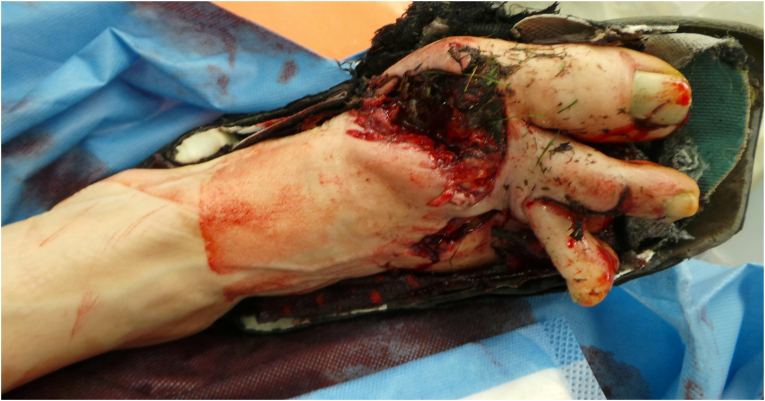
Dorsal view of the affected foot with heavily contaminated wounds at initial presentation.

**Fig. 7:**
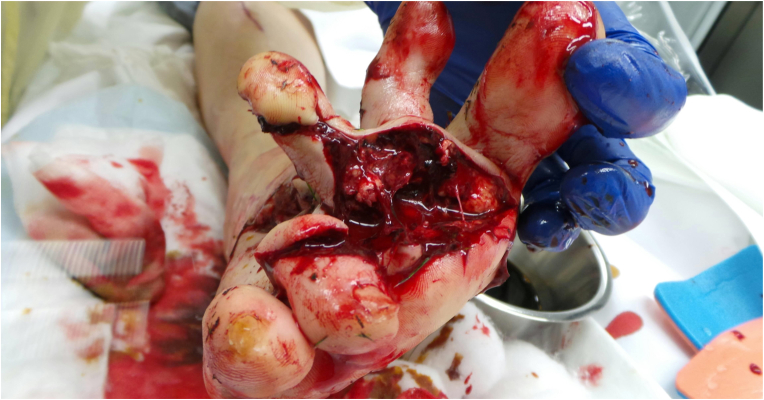
Plantar view, further illustrating the extent of tissue damage.

This 64 years old woman injured her right foot in a lawnmower accident, sustaining multiple 3° open fractures of the metatarsal bones I-IV, including significant bone loss in the first metatarsal ray (Fig. 5). Both the dorsal and plantar wounds were heavily contaminated with partially degloved soft tissues (Fig. 6, Fig. 7), which resulted in a significant skin defect after debridement, especially over the first metatarsal and medial aspect of the foot.

**Fig. 8:**
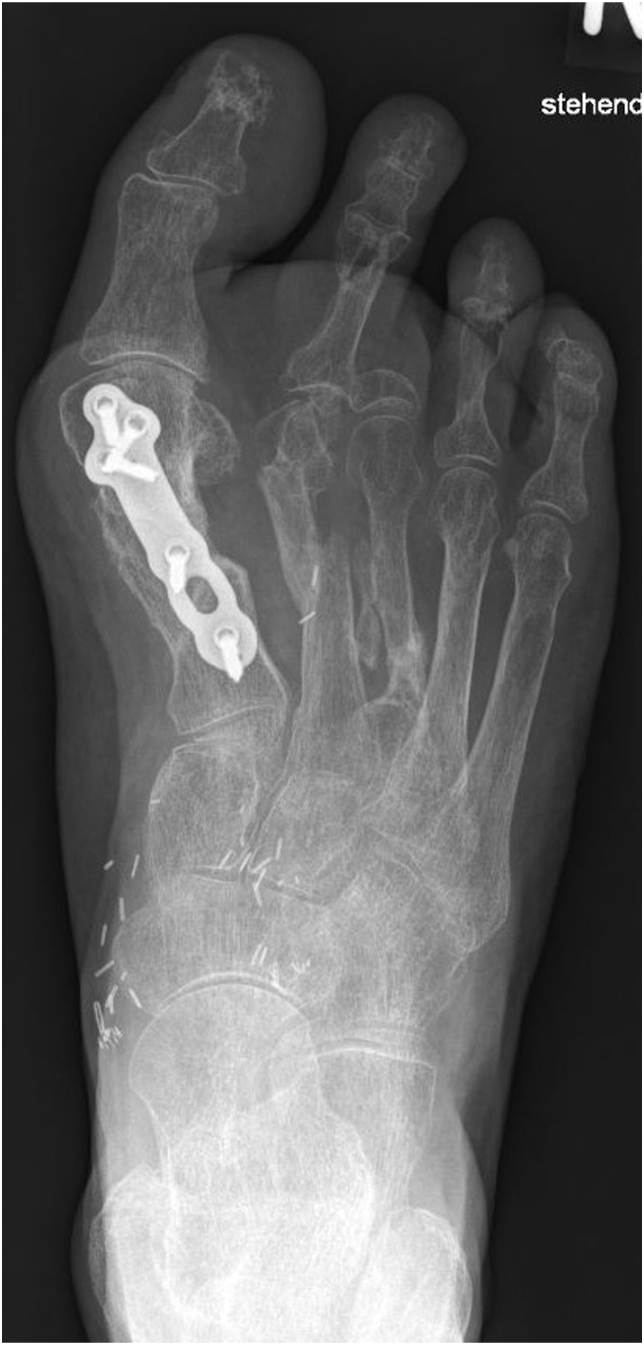
Radiological follow-up 10 months postoperative illustrating complete bony union of the first metatarsal.

**Fig. 9:**
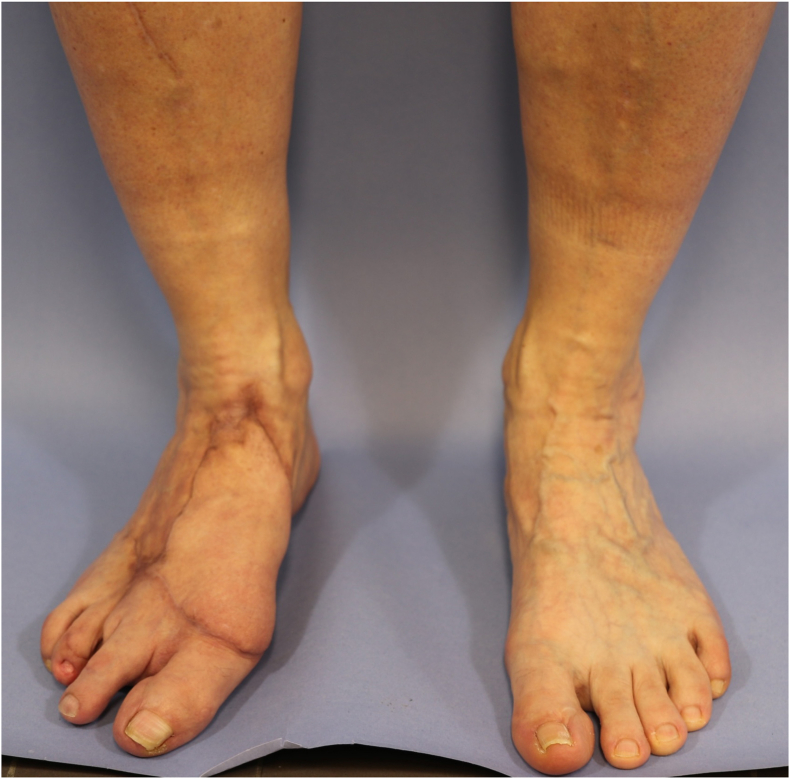
Clinical result 14 months after the reconstruction.

The case was discussed in an orthoplastic interdisciplinary team with the orthopaedic and trauma surgeons. The third toe was avascular and was therefore resected. In order to provide both vascularized bone for the reconstruction of the osseous defect of the first metatarsal ray as well as thin soft tissue coverage in one procedure, an osteocutaneous SCIP flap was chosen. Reconstruction was performed nine days after the initial trauma. The skin paddle used measured 13 × 7 cm, while a 4 × 2 cm block of vascularized bone was necessary to bridge the osseous defect. The flap was anastomosed end-to-side to the dorsalis pedis artery and healed without the need for any secondary interventions. Fig. 8, Fig. 9 illustrate both a radiographic follow-up 10 months postoperative with complete bony integration, as well as the clinical findings roughly 14 months after the reconstruction. Long-term follow-up at 36 months shows that the patient can put her full body weight on the foot, walk longer distances, and does not wish for any secondary corrections.

## 4 Discussion

Microsurgical reconstruction of composite bone and soft tissue defects in the extremities remains a complex challenge, requiring careful preoperative planning and individualized decision-making. The choice of flap must be tailored to the specific defect characteristics, considering all available reconstructive options, surgeon's expertise, donor site morbidity, as well as patient-specific factors such as comorbidities or body habitus.

The osteocutaneous SCIP flap offers several advantages in this context. It provides a reliable and customizable soft tissue component with a generally low donor-site morbidity and minimal functional impairment. However, a key limitation of this flap lies in the relatively restricted volume of vascularized bone that can be harvested. The exact extent of the bone segment that is vascularized by the SCIA system remains an issue of debate. While clinical and cadaveric evidence suggests that bone segments measuring up to 8 × 3 cm are reliably perfused, the dimensions certainly vary depending on the individual's vascular anatomy.^20^^,^^21^ Intraoperative assessment of bone perfusion - through observation of bleeding at the osteotomy sites or indocyanine green (ICG) fluorescence angiography - therefore remains key for ensuring viability of the osseous component.

In comparison to this, the fibula flap enables harvest of significantly larger bone segments while also offering a dependable skin paddle.^22^ However, the soft tissue component is less independent from the bone due to its septocutaneous attachments, limiting flexibility of inset. Furthermore, a sizable skin island usually necessitates split-thickness skin grafting to close the donor site, which increases overall donor site morbidity. Moreover, the flap also requires the patient to sacrifice one of the three major vessels of the lower leg.

The deep circumflex iliac artery (DCIA) flap similarly allows for harvest of a more substantial bone segment, but its bulky, often unreliable skin paddle and higher incidence of donor site complications such as abdominal wall herniation have dampened its popularity.^23^

Meanwhile, alternatives like the osteocutaneous radial forearm flap, the medial femoral condyle flap or the lateral arm flap are better suited for small defects, as the volume of vascularized bone that can safely be included is rather limited. The osteocutaneous SCIP flap may therefore occupy a unique niche in microsurgical limb reconstruction, especially in cases in which a moderately sized vascularized bone segment is required in addition to thin soft tissue coverage.

**Fig. 10:**
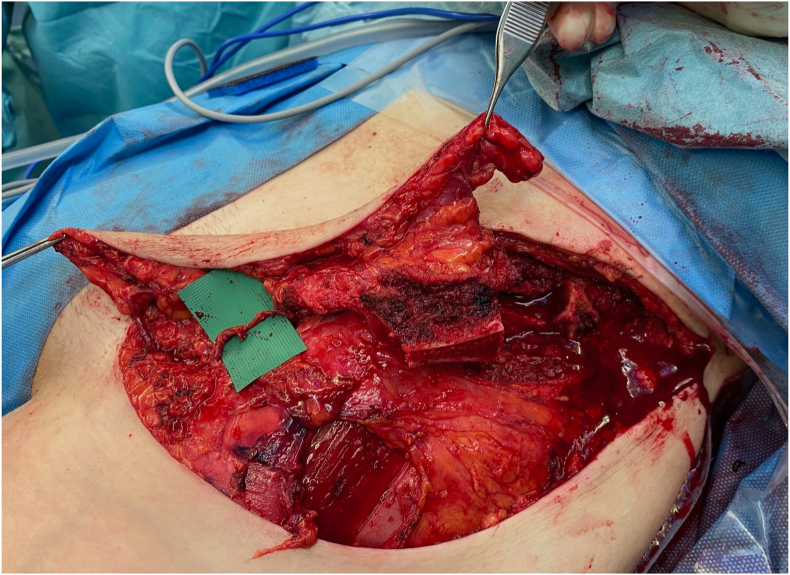
Example of an osteocutaneous SCIP flap raised in a composite fashion. The green microsurgical background was placed under the deep branch of the SCIA for better visualization.

**Fig. 11:**
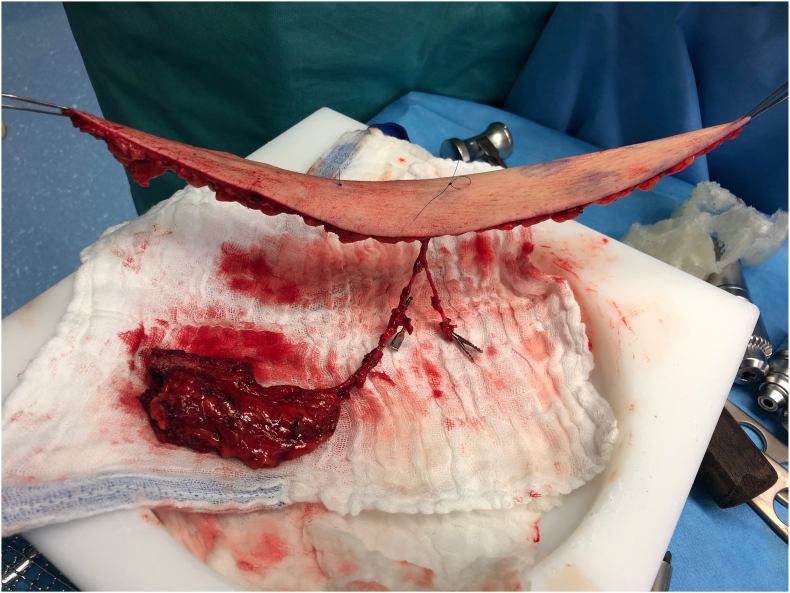
Osteocutaneous SCIP flap raised in a chimeric design, allowing for more independent inset of the bony and soft tissue components. The picture also highlights how thin the skin island can be harvested if no direct attachment to the bone is necessary.

There are different techniques of how an osteocutaneous SCIP flap can be designed and harvested. Both the superficial and deep branch can be included, and the flap can be raised either in a composite fashion (Fig. 10), leaving the bone component attached to the overlaying skin, or as a chimeric flap. In general, we prefer a chimeric deep branch only design as previously described.^18^ The independency of the skin island and bone segment greatly simplifies both the inset and osteosynthesis at the recipient site. Furthermore, it allows the surgeon to harvest the soft tissue component of the flap on a more superficial level, resulting in a thinner flap. This consideration may be particularly relevant in Western populations, where a higher prevalence of overweight individuals is observed (Fig. 11). However, this design relies on the presence of a sizable bone branch originating from the deep branch. As we start flap elevation caudally, the deep branch perforators to the skin are encountered first. Tracing backwards from them, the presence of the bone branch can be verified. If no significant single branch can be identified, dissection is continued in a composite fashion, mobilizing the bone while left completely attached to the skin island.

With both techniques, the anterior superior iliac spine is left in place, but the periosteum on top of it is harvested with the flap to preserve the bone branch running along the crista.

**Fig. 12:**
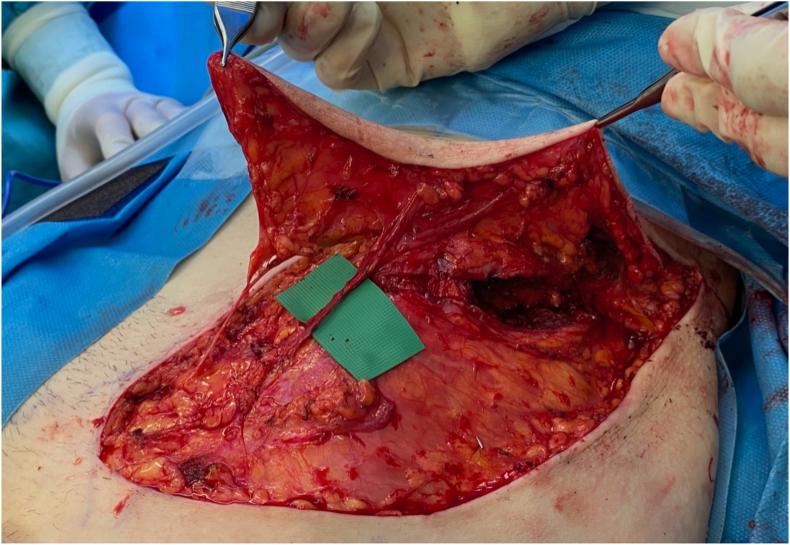
Osteocutaneous SCIP flap being raised. The green microsurgical background was placed under the deep branch of the SCIA to illustrate the pedicle length that can be achieved using this branch, even before opening the deep fascia and dissecting back to the origin from the femoral vessels.

Another advantage of the deep branch only design is the gain in pedicle length. With a mean length of 9.1 ± 1 cm if the flap is raised on the superficial plane, the deep branch offers a significantly longer pedicle compared to the superficial branch (Fig. 12).^5^

Regarding the arterial anastomosis we usually prefer end-to-side anastomosis in extremity reconstruction, either directly onto a main vessel, or functionally by using the stump of a side branch of one of these main vessels end-to-end. On one hand, this approach preserves the perfusion of the limb distal to the defect. On the other, given the relatively small mean diameter of the arterial pedicle (2.3 mm on average), it also mitigates the challenge of a significant size mismatch between the recipient vessel and the SCIA.

**Fig. 13:**
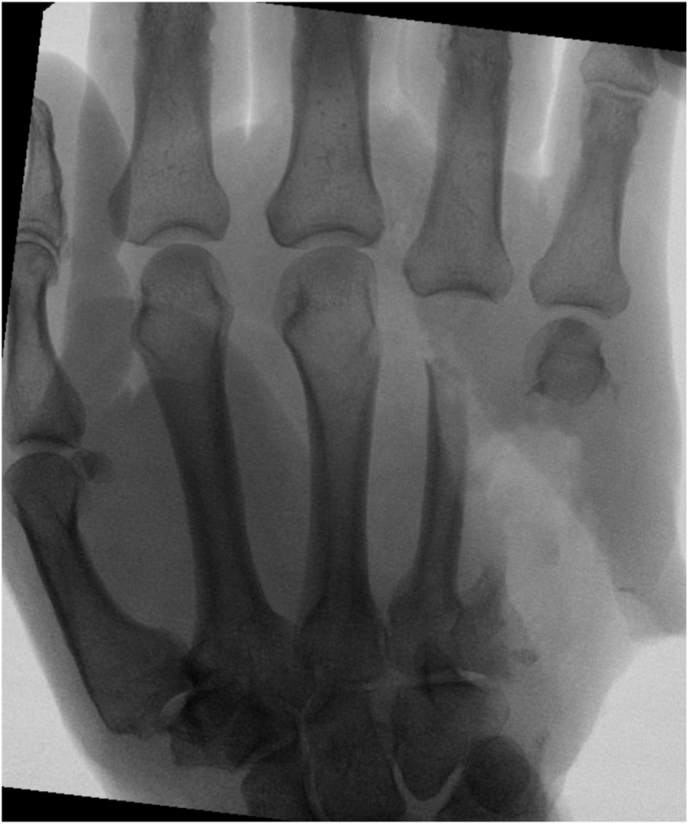
Initial presentation after circular saw injury resulting in an almost complete destruction of the 5th metacarpal bone as well as the distal third and head of the 4th metacarpal.

**Fig. 14:**
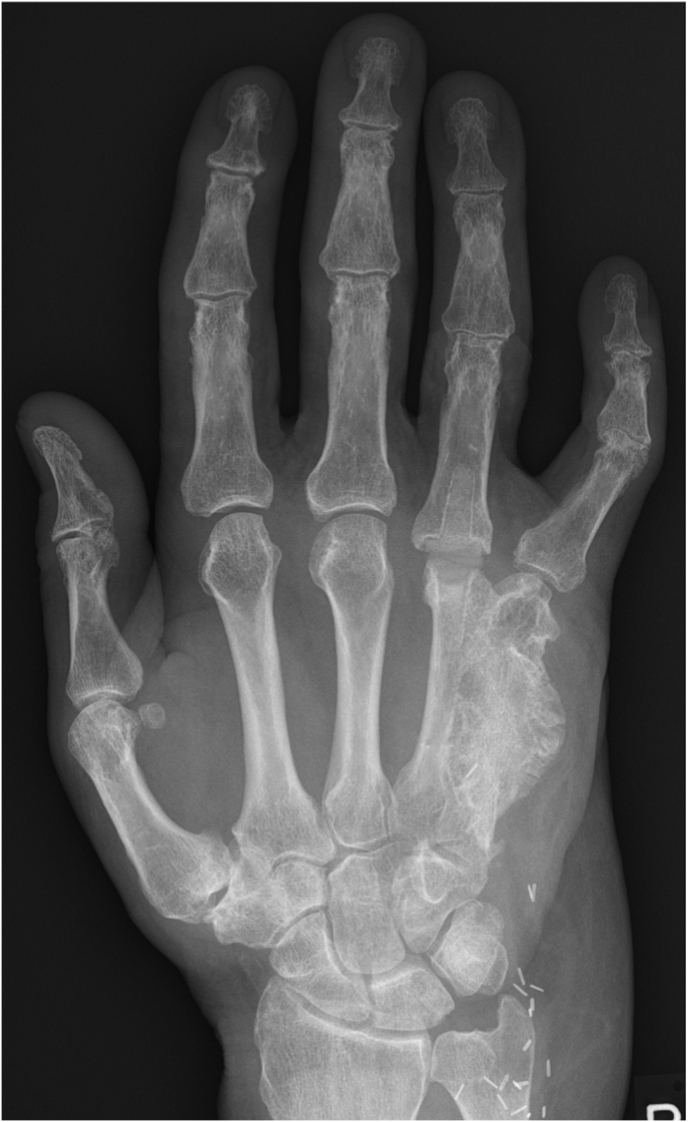
Follow-up radiograph two years after reconstruction of the 5th metacarpal and distal part of the 4th metacarpal with an osteocutaneous SCIP flap. One year after the initial trauma an MCP 4 arthroplasty was performed, placing a KeriFlex (KeriMedical, CH) implant into the completely consolidated iliac bone stock.

Although the size of the bone segment that is vascularized by the SCIA is limited as discussed above, the quality of the bone is excellent and has therefore been used for a long time in a wide range of bony reconstructions, especially as non-vascularized grafts. If a full thickness segment of the iliac crest is harvested, the block consists of tricortical bone, which provides immediate stability and allows for early weight bearing, for example after reconstruction of tibial defects, while also serving for osteoconduction. Meanwhile, the included trabecular bone and bone marrow provide osteoinduction and osteogenesis. The quality and tricortical nature of the bone also enables placement of osseointegrated implants (see Fig. 13, Fig. 14). In fact, implant stability following placement in iliac crest bone appears to be comparable to that achieved with fibula flaps.^24^

In our series, complete primary bony union was achieved in 8 of 9 cases (89 %) after a mean postoperative follow-up of 8.25 months, with only one patient developing pseudoarthrosis at one of the two docking sites. This is comparable to the existing literature regarding both the time to union as well as the union rate achieved with other free vascularized bone flaps. Previous studies on the osteocutaneous SCIP flap have documented a time to bony union ranging from 5 to 9 months.^20^^,^^25^

**Fig. 15:**
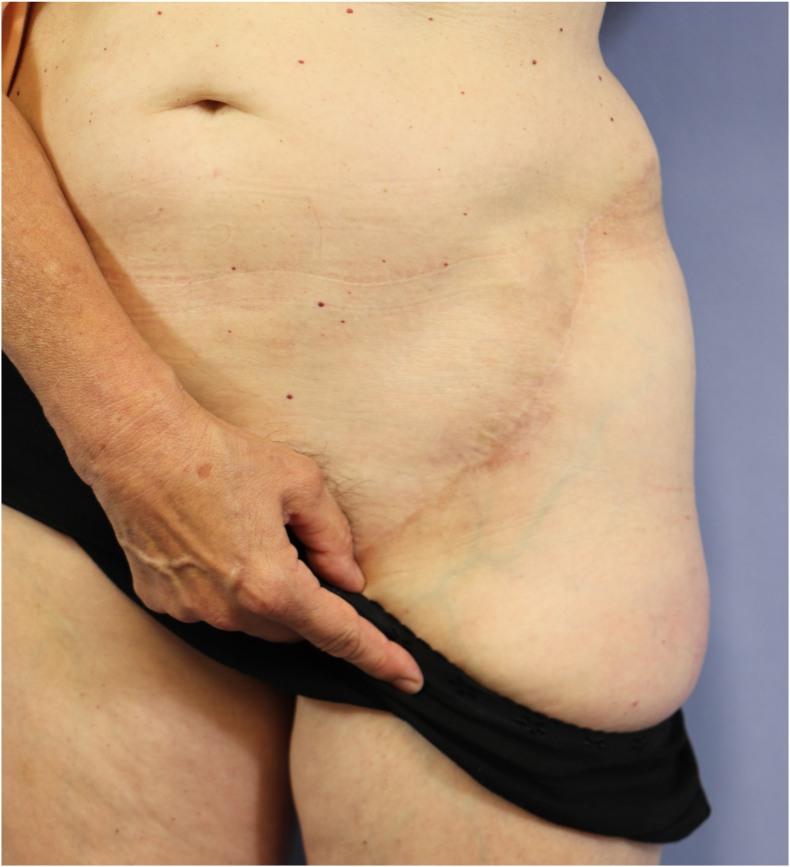
Example of a donor site scar after harvest of an osteocutaneous SCIP flap.

**Fig. 16:**
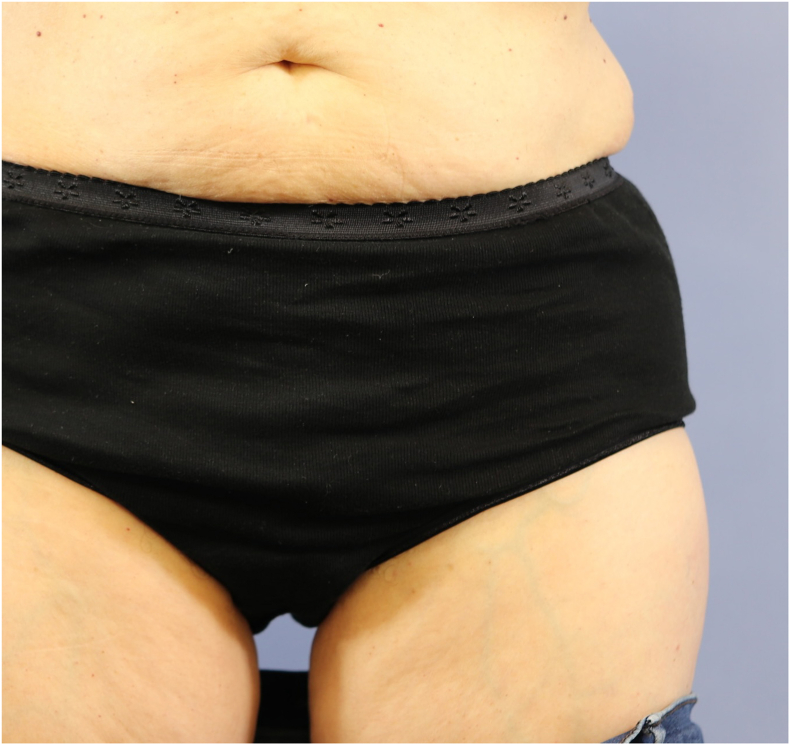
The donor site scar after harvest of an osteocutaneous SCIP flap is easily concealed.

The SCIP flap is generally hailed for its low donor site morbidity. The scar in the inguinal region is inconspicuous and easily concealed by underwear or swimwear in both males and females (Fig. 15, Fig. 16). However, harvesting bone from the iliac crest as part of the flap of course adds a certain amount of morbidity specific to this part of the procedure, most commonly a contour defect and postoperative pain.^25^ We attempt to reconstruct the contour defect of the iliac bone, especially in case of larger bone segment harvests by using bone allografts (Tutoplast®) fixed in the defect with a press-fit technique and covered with a resorbable mesh attached via sutures and drill holes in the inner and outer iliac cortical bone.

**Fig. 17:**
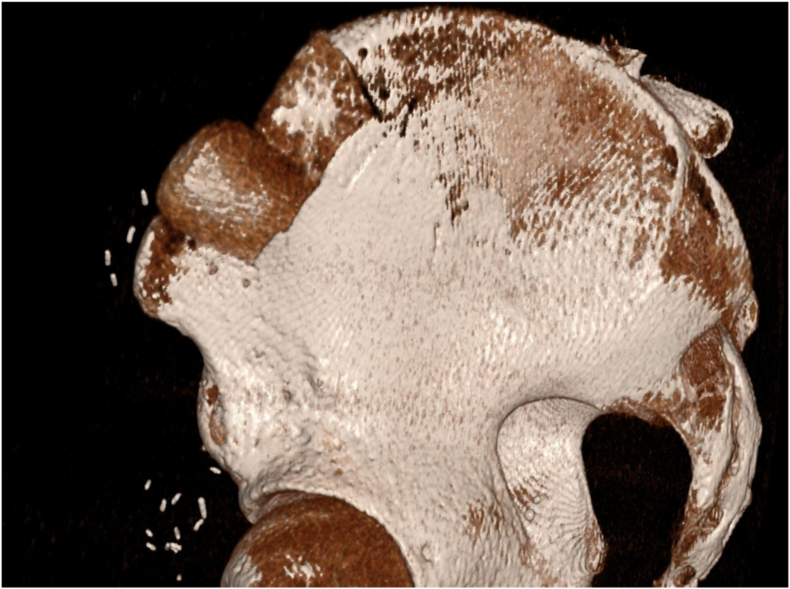
Early postoperative CT of an osteocutaneous SCIP donor site with bone allografts (Tutoplast®) in place. Additionally, the iliac wing fracture starting from the donor site can be seen.

Unfortunately, one of our cases suffered a non-displaced iliac wing fracture, which healed uneventfully under conservative treatment (Fig. 17). Fractures of the iliac crest are a known complication after any kind of iliac bone harvest, whether vascularized or non-vascularized.^26^ Most of these can be treated conservatively, especially if bone graft harvesting is performed from the anterior region such as in the case of an osteocutaneous SCIP flap, whereas bone harvesting from the posterior iliac crest seems to be prone to more severe complications.^27^

At the recipient site, two patients developed a hematoma, which was treated by surgical drainage in the early postoperative phase. Although re-exploration did not reveal any definitive underlying cause for this finding, we assume that it may be a complicating factor of the bony reconstruction. During the surgery, shortening osteotomies are performed at the recipient site until only well perfused bone remains, after which the iliac bone segment is trimmed and inset, adding at least another three osteotomy sites, all of which can add to diffuse bleeding. Furthermore, the skin island is usually designed to be very thin. While this avoids excessive bulk and improves the cosmetic outcome of a one-stage procedure, any amount of fluid may also be more conspicuous underneath. We have not observed a similar trend concerning hematoma formation with any of our other SCIP flap cohorts that did not include osseous reconstruction.

We acknowledge that this study has several limitations. First, the retrospective design inherently introduces the risk of selection bias and limits the control over confounding variables. Second, despite representing the largest dedicated series on the osteocutaneous SCIP flap for extremity reconstruction in the western population, the sample size is relatively small. Third, the absence of a control group limits the ability to directly compare the SCIP flap to other osteocutaneous options such as the fibula, DCIA, or medial femoral condyle flaps. And lastly, the complex injury patterns in the upper extremity cases were too diverse to extract any meaningful uniform outcome data.

## 5 Conclusions

The osteocutaneous SCIP flap is a viable and versatile option for orthoplastic reconstructions of complex extremity defects requiring both soft tissue and vascularized bone. The flap shows reliable vascularity, provides a bony union rate comparable to other vascularized bone flaps and the donor site scar is easily concealed. We therefore advocate for greater consideration of this flap, particularly in cases requiring a moderately sized vascularized bone segment as well as by thin, pliable soft tissue coverage. Its versatility makes it a valuable addition to the reconstructive repertoire, especially for addressing defects in the distal regions of both the upper and lower extremities.

## Author contributions

Conceptualization: RO, CZ, CO; Data curation: CZ; Formal analysis: CZ; Funding acquisition n/a; Investigation: RO, CZ; Methodology: RO, CZ, IL; Project administration: RO, CZ; Resources: CZ; Software: RO, CZ; Supervision: RO, CO; Validation: RO, CZ; Visualization: RO, CZ; Roles/Writing - original draft: CZ; Writing - review & editing: RO, CZ, IL.

## Funding

This research did not receive any specific grant from funding agencies in the public, commercial, or not-for-profit sectors.

## Declaration of competing interest

The authors declare that they have no known competing financial interests or personal relationships that could have appeared to influence the work reported in this paper.
